# Seminal Plasma Metabolomic Responses to Selenium Supplementation and Testicular Heat Stress in Zulu Rams

**DOI:** 10.3390/cells15201901

**Published:** 2026-10-19

**Authors:** Khomotso Podile Molvia Lekola, Mbukeni Andrew Nkomo, Khoboso Christina Lehloenya

**Affiliations:** 1 Department of Agricultural Economics and Animal Production, University of Limpopo, Polokwane 0700, South Africa; 2 School of Science and Agriculture, University of KwaZulu-Natal, Durban 4000, South Africa; nkomom@ukzn.ac.za; 3 Department of Agriculture, University of Zululand, Empangeni 3880, South Africa; lehloenyak@unizulu.ac.za

**Keywords:** fertility biomarker, seminal plasma, selenium, metabolomics, testicular heat stress, Zulu rams

## Abstract

**Highlights:**

**What are the main findings?**

- The study identified a total of 536 metabolites; 32 metabolites were found to be unique to each treatment (9—control; 6—selenium; 6—heat stress; 11—heat stress + selenium).
- Over 50% of the metabolites identified in the study were not characterized.

**What are the implications of the main findings?**

- Selenium supplementation affected metabolites involved in pathways related to pyrimidine metabolism, fatty acid biosynthesis, glycine, serine, and threonine metabolism, aminoacyl-tRNA biosynthesis, glutathione metabolism, alanine and aspartate metabolism, branched-chain amino acid metabolism, and the tricarboxylic acid (TCA) cycle.
- The uncharacterized metabolites were a major limitation in the study and their role in seminal plasma following selenium supplementation is unclear.

**Abstract:**

Selenium plays an essential role in animals because of its antioxidant properties and direct influence on reproductive health, which improves sperm quality for maximum fertility. This study aimed to investigate the untargeted metabolomic profiling of seminal plasma of Zulu rams exposed to heat stress and selenium supplementation. For this, seminal plasma (SP) was harvested from semen (stored at −80 °C) collected from 20 Zulu rams aged 2–5 years that were randomized into four groups according to their body weight and scrotal circumference: selenium with testicular heat stress; G1, selenium; G2, control; G3, and 6 weeks of exposure to testicular heat stress; G4. Untargeted spectrum data were analyzed using MetaboAnalyst, and normalized to ensure reproducibility and reliability, and quality control samples were generated by mixing small aliquots of each analytical sample to assess metabolite variance. The study identified a total of 536 metabolites potentially linked to fertility, over 50% of which were unclassified. Of the 536 metabolites, 359 were conserved across all treatment groups. Selenium supplementation resulted in a reduction in the abundance of several metabolites involved in pathways of pyrimidine metabolism, fatty acid biosynthesis, glycine/serine/threonine metabolism, and aminoacyl-tRNA biosynthesis. The supplementation of selenium for rams under heat stress conditions altered pathways related to branched-chain amino acid metabolism, alanine/aspartate metabolism, and glutathione metabolism. Conversely, heat stress conditions without selenium supplementation reduced pathways associated with caffeine metabolism, fatty acid degradation, pyrimidine metabolism and flavonoid biosynthesis. The study was able to reveal seminal plasma metabolites induced by selenium supplementation and heat stress conditions. However, the study faced a major limitation of a high proportion of uncharacterized metabolites.

## 1. Introduction

The Republic of South Africa (RSA) is a hotspot for animal genetic biodiversity, including indigenous sheep breeds such as the Damara, Namaqua Afrikaner, Pedi, and Zulu, which play a pivotal role in the livelihoods of smallholder farmers in rural areas [1]. Many of these sheep breeds are facing extinction due to inbreeding and unplanned breeding with exotic breeds [2]. Thus, conservation efforts are crucial, particularly for indigenous breeds like the Zulu ram, which are highly tolerant to diseases and parasites. According to Fitzgerald and Morgan, the ram can sire between 150 and 300 lambs in his lifetime as opposed to a doe which typically gives birth 4 to 6 times in her lifetime [3]. Thus, the ram has the largest influence on herd fertility and serves as a primary driver of genetic quality, population health and reproductive performance. Therefore, it is important to know the fertility of a breeding ram for conservation purposes and for repopulation of a breed. These breeds are essential for ensuring stability and continuity in modern livestock production [2].

Semen quality parameters such as progressive motility, concentration, morphology and DNA integrity are used in several studies to predict fertility [4,5,6]. Therefore, evaluating semen quality parameters is a good starting point for understanding the fertility of a breed such as the Zulu sheep breed. The study by Chella is by far the only study that analyzed the influence of season in semen quality parameters of Zulu rams found in the KZN province [7]. The study found that Zulu ram semen quality was low in summer as opposed to autumn, winter and spring. Although the study by Chella did not attribute the poor semen quality to heat stress (HT) that could emanate from the high ambient temperatures experienced in summer, it could be a possible contributing factor. This is because spermatogenesis is generally favoured when scrotal temperature remains 4–6 °C below core body temperature, which is typically maintained under ambient temperatures of approximately 20–30 °C [8]. When ambient temperatures rise above 20–30 °C, exceeding the thermoneutral zone for rams, testicular thermoregulation is activated to maintain the testes at a lower temperature [9]. However, if the heat load continues and ambient temperatures reach around 32 °C or higher, heat stress can raise body and scrotal temperature enough to reduce sperm production, sperm motility and increase abnormal morphology [10].

The KZN province, which is home to the Zulu ram, has been experiencing heatwaves, reaching a high of 37–40 °C in summer [11]. These temperatures experienced in KZN are well above the thermoneutral zone for rams, which may explain why Chella observed poor semen quality parameters during summer. A study by Lekola used seminogram analysis and found that induced HS reduced the quality of certain sperm parameters in Zulu ram semen [12]. The study further observed that selenium distinctively improved certain sperm quality parameters of Zulu rams subjected to HS. Therefore, the study aims to understand the metabolites and associated pathways induced under selenium supplementation and induced testicular HS.

## 2. Materials and Methods

### 2.1. Animals Management and Experimental Design

All animal care and procedures used were performed in accordance with the Animal Ethics Committee of the University of Zululand (UZREC Project no: 17111-030 PGD 2021/20). The present study was carried out at the University of Zululand Dairy farm in Dlangezwa, over a period of three months towards the end of summer and autumn (February to May 2022). A total number of 20 Zulu rams aged between 24 and 60 months were grouped according to body weight and scrotal circumference. The rams were separated from the ewes, allocated randomly into four groups, comprising 5 animals per group. The animals were raised only on locally available milled lucerne weaning free of selenium (purchased from Northern Cape, Jankempdorp, South Africa) and they had no access to fresh growing forages or other feed for four months before the start of the experiment following a procedure described by Lukusa and Lehloenya [13]. For experimental design, the treatment groups were arranged as follows: G1 represents seminal plasma from rams treated with selenium and induced testicular heat stress (SeTHS); G2 is seminal plasma from rams treated with selenium only (Se); G3 is seminal plasma from the control group (C: no selenium or induced heat stress) and lastly G4 is seminal plasma from rams with induced heat stress only (THS). The rams subjected to dietary selenium were given sodium selenite (Sigma Aldrich, Merck KGaA, Darmstadt, Germany) orally at a dosage of 0.34 Se mg/kg of body weight. Dietary selenium was administered once a week for a period of 4 months. Heat stress was induced by wrapping a thermal testicular insulation bag around the scrotum of the rams. The rams wore the insulation bags intermittently over a period of 49 days, alternating between one week of wearing the bags and one week of rest. This period covered the first spermatogenesis cycle. During the subsequent 4-month data collection period, the rams did not wear the insulation bags to prevent permanent damage to the Sertoli cells and scrotal tissue.

### 2.2. Semen Collection and Seminal Plasma Harvesting

Semen was collected once per week for a period of three months using an EL Toro 3 electro-ejaculator (Electronic Research Group, Halfway House, Midrand, RSA) with the mode set to 3–6 volts for 3 s at 7-s intervals following a modified protocol adapted from Lukusa and Lehloenya [13]. The first month of the four-month period was dedicated to selenium acclimatization. Prior to semen collection, the sheath covering the penis was cleaned with a sterile towel containing 70% ethanol. The rectal probe was lubricated and inserted into the rectum above the accessory sex glands for stimulation. Four levels of 30 volts were applied through the probe to induce ejaculation. Semen was collected using pre-warmed 15 mL tubes and immediately taken to the laboratory for analysis of semen and sperm quality parameters. Seminal plasma was harvested by centrifugation at 2000 rpm for 13 min. The supernatant was centrifuged again to remove excess sperm cells. Seminal plasma was then transferred to 1.5 mL Eppendorf tubes (Merck KGaA, Darmstadt, Germany) and stored at −80 °C for further metabolomic analysis.

### 2.3. Reagents and Chemicals

BSTFA + 1% TMCS (N,O-Bis(trimethylsilyl)trifluoroacetamide with trimethylchlorosilane) was purchased from Sigma-Aldrich (St. Louis, MO, USA). All organic solvents used were ultra-pure Burdick & Jackson brands (Honeywell International Inc., Muskego, MI, USA).

### 2.4. Metabolite Extraction and Derivatization for GC-TOF/MS Analysis

Seeing that the amount of seminal plasma necessary for GC-MS analysis is standardized, individual samples were processed following a modified method by Yi et al., 2014 [14]. Each seminal plasma sample (100 µL) was spiked with 100 µL of 3-phenyl butyric acid (100 ppm) as an internal standard. Proteins were precipitated by adding 300 µL of ice-cold methanol to each sample. The samples were then incubated on ice for 10 min to ensure complete protein precipitation. Following incubation, the samples were centrifuged at 12,000 rpm for 8 min at 4 °C. The supernatants were carefully collected to avoid disturbing the precipitate. The collected supernatants were dried under a stream of nitrogen gas. The dry extracts were then derivatized by adding 50 µL of methoxyamine hydrochloride in pyridine (15 mg/mL) and incubating at 50 °C for 90 min. Subsequently, 40 µL of N,O-Bis(trimethylsilyl)trifluoroacetamide containing 1% trimethylchlorosilane (TMCS) was added, and the samples were incubated at 60 °C for 60 min.

### 2.5. Peak Identification

Peak identification in the study was done following Schymanski et al. (2014) [15]. ChromaTOF v.4.50 software (Leco Corporation, St. Joseph, MI, USA) for peak finding and mass spectral deconvolution at a signal-ratio of 200, with a minimum of three apexing peaks. Mass fragmentation patterns generated by MS, together with their respective GC retention times, were compared to commercially available National Institute of Standards and Technology spectral libraries, (mainlb, replib; https://chemdata.nist.gov/). A similarity threshold of 800 (80%) required for a name to be assigned to a peak.

Raw GCxGC-MS data, including peak areas and annotations for each sample, were exported to Ms excel. Compounds with the same name and unique mass (UM) were consolidated. Contaminant compounds were identified by comparing the compounds detected in the extraction blank and system blank to that detected in the samples. Peaks detected in the system blank were deleted from the database if their average concentration in the samples was 20 times or less than the average in the system blanks. Compounds present at concentrations 20 times or higher in the average of samples, compared to the system blanks, were kept in the data set and coloured green. Peaks detected in the extraction blanks were deleted from the database if their mean concentration in the sample was 3 times or less than that in the blanks. Samples were quantified using the detected area of the internal standard to get to a relative concentration in ng per sample.

### 2.6. Statistical Analysis

We used statistical multivariate analysis and machine learning methods to build prediction models using serum metabolites, and we plotted a Venn diagram for control, selenium, heat, and heat + selenium groups. Data normalization was performed using median fold normalization, and the data were log-transformed because they were not normally distributed, and different metabolites with large differences in concentration were analyzed together. For univariate analysis, such as Student’s *t*-test, we used non-normalized and non-transformed data (raw data). This comparison was done for control versus selenium treatment, aiming to identify selenium-induced metabolites under normal conditions. Additionally, we compared heat stress (heat) versus heat stress + selenium (heat + selenium) to determine selenium-induced metabolites under heat stress. MetaboAnalyst 4.0 (www.metaboanalyst.ca, accessed on 27 April 2023) and Funrich 3.1.3 (https://www.funrich.org/download#, accessed on 13 February 2024) were used for multivariate and univariate analysis. The area under the receiver operating curve (AUC), sensitivity, and specificity were obtained using a multivariate approach.

## 3. Results

The results were captured and presented, confirming the accurate identification of the compounds. A total of 536 metabolites were identified, while a total 164 metabolites were assigned names, and 372 metabolites remained unclassified.

This study also explored the impact of selenium (Se) on the seminal plasma metabolome under induced testicular heat stress in Zulu rams, aiming to deepen our understanding of the metabolic mechanisms underlying Se-mediated heat stress resilience.

**Figure 1:**
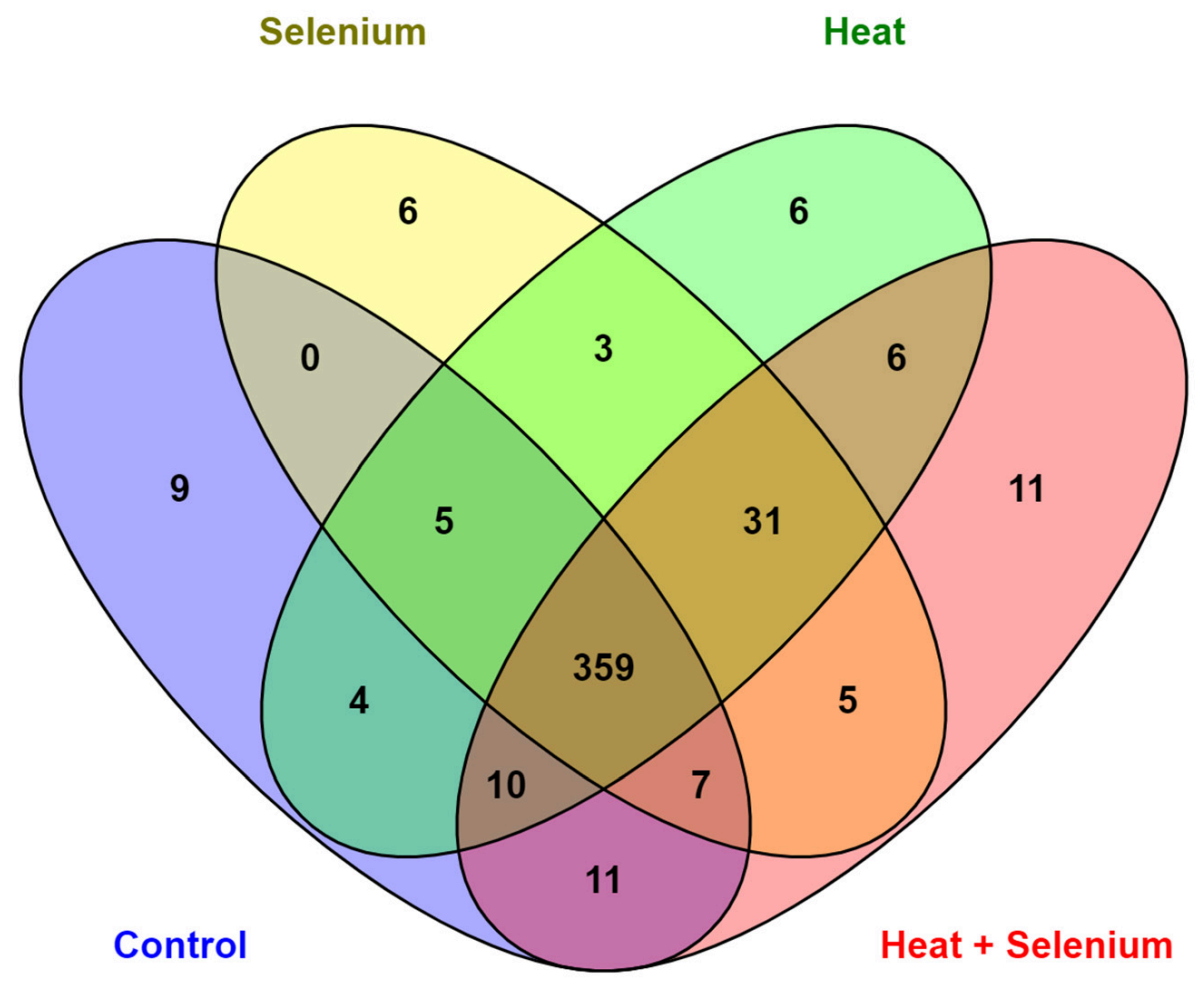
Detection of selenium responsive metabolites on Zulu rams’ seminal plasma exposed to testicular heat stress. Venn diagram illustrating the common and unique metabolites identified in all treatments (control, selenium, heat stress, and heat stress + selenium). The numbers within each section represent the count of unique or shared metabolites, with the corresponding metabolite’s identifications indicated in parentheses. The central section shows 359 metabolites out of 536 were conserved across all treatments.

To achieve this, untargeted metabolomics analysis in our current study identified a total of 536 metabolites across all treatments. The 536 metabolites that were identified in this study were analysed using the FunRich Multi analysis software package (version 3.1.3), to identify common and unique metabolites in each of the different treatments (Figure 1). The results showed that 359 of 536 metabolites identified in this study were conserved across all treatments. On the other hand, 32 metabolites were found to be unique to specific treatments (9—control; 6—selenium; 6—heat stress; 11—heat stress + selenium).

### 3.1. Identification of Metabolites

**Figure 2:**
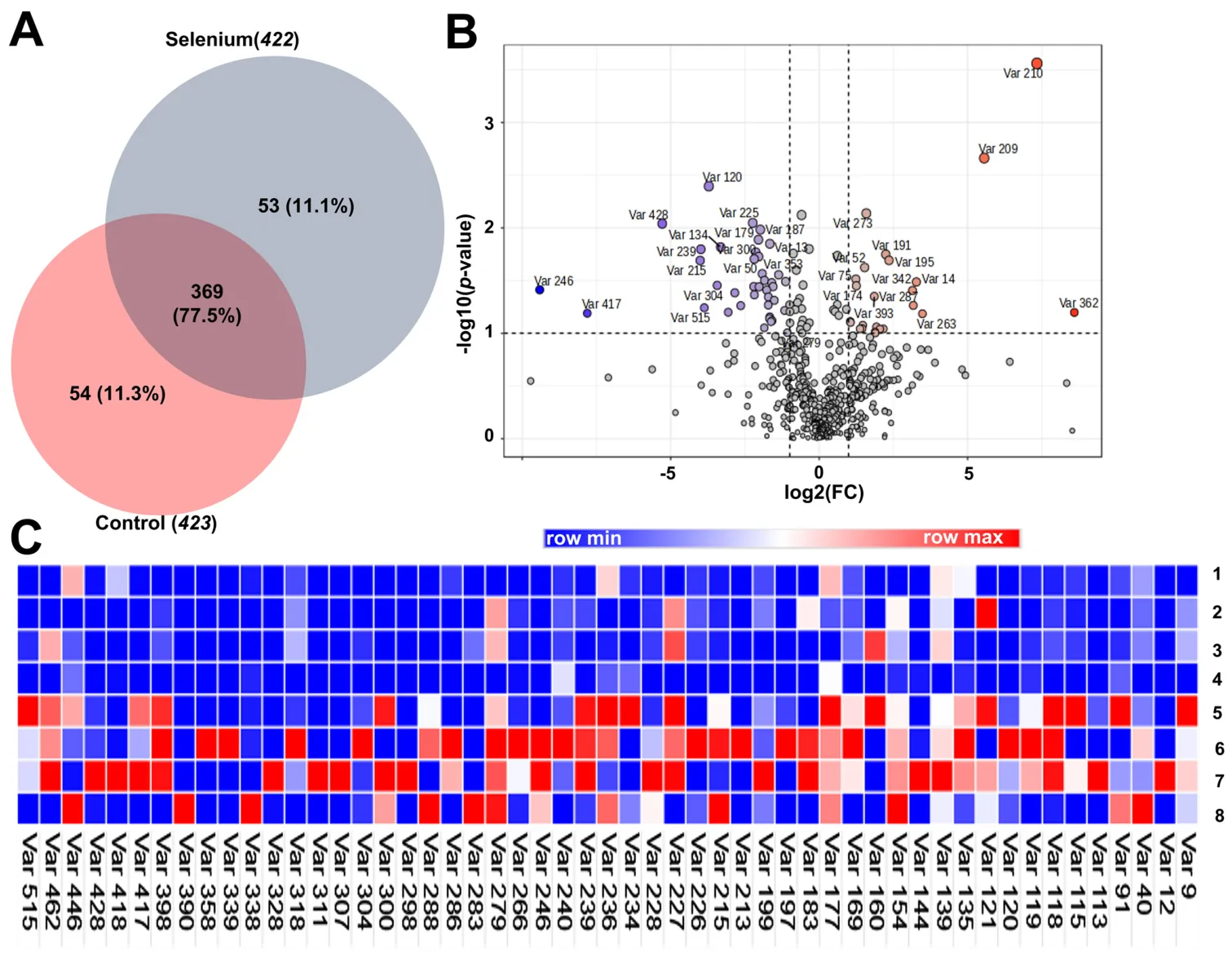
Metabolomic profiling of seminal plasma from Zulu rams supplemented with or without selenium on their feed. (**A**) Venn diagram illustrating the overlap of detected metabolites between control (pink) and selenium-supplemented (grey) groups. Shared metabolites (n = 369, 77.5%) represent common metabolic pathways active under both conditions, while unique metabolites in the control (n = 54, 11.3%) and Selenium (n = 53, 11.1%) groups suggest metabolic shifts induced by selenium supplementation. (**B**) Volcano plot of differential metabolite expression between control and selenium groups. Each point represents a metabolite, with red points marking significantly upregulated metabolites in the selenium group and blue points marking those downregulated compared to the control group (thresholds for significance are set at *p* < 0.05 and |log2 fold-change| > 1). Metabolites with no significant change are shown in grey/black. This plot identifies key metabolic alterations associated with selenium supplementation. (**C**) Heatmap of unnormalized metabolite intensities, of the 53 selenium-induced metabolites identified in Figure 2A. Each row represents a metabolite, and each column represents an individual sample. Relative intensity is depicted on a colour scale, with red indicating high abundance (row max) and blue indicating low abundance (row min).

The Venn diagram in Figure 2A provides valuable insight into the categorization of metabolites, particularly when comparing the control group to the selenium-treated group. The significant overlap in metabolite profiles underscores the shared metabolic pathways active across these conditions, while the presence of distinct metabolites highlights potential unique biochemical responses or adaptations specific to selenium supplementation. Notably, metabolites such as Var 210 and Var 209 show significant upregulation in the volcano plot, indicating an inducement by selenium supplementation. The heatmap further illustrates variations in metabolite abundance of the 53 unique metabolites, with distinct indications of differential responses to selenium supplementation. While 51 of the 53 metabolites remained unknown, meaning that future studies should focus on annotating and experimentally validating the unknown metabolites, particularly those with significant differential expression, such as Var 210 and Var 209.

### 3.2. Quantification of Selenium Induced Metabolites in the Present and Absent of Heat

For control vs. selenium samples a total of 476 metabolites were identified as true metabolites passing the threshold criteria (including 423 out of 536 in control and 422 out of 536 selenium), and 369 metabolites (77.5%) were conserved between the control and selenium treatment, while 53 unique selenium-induced metabolites (11.1%) were identified in the selenium-only treatment samples (Figure 2A,C; Table 1). Further analysis of the 53 unique metabolites in Table 1; 84.4% were unclassified, while sugar and amino acid derivatives both shared 6.7% each and the remaining 2.2% belonging to organic acid (Table 1). Further analysis using a volcano plot to detect the expression levels of metabolites revealed that from the upregulated metabolites six were found to be induced only by supplementation of selenium (Table 1).

### 3.3. Metabolites Distribution of the Seminal Plasma Metabolome

**Figure 3:**
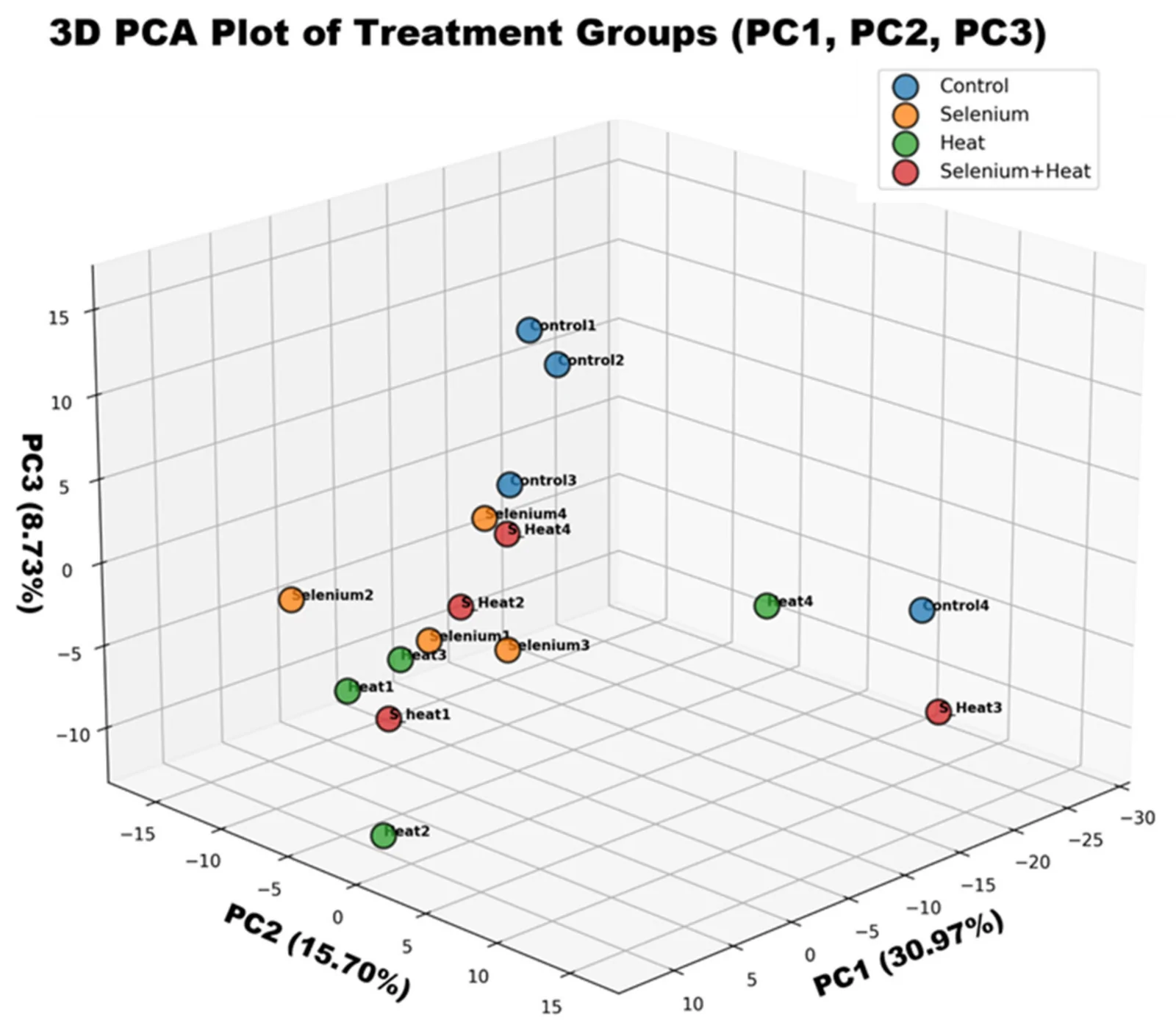
Principal Component Analysis (PCA) of seminal plasma metabolites in Zulu rams subjected to selenium supplementation and testicular heat stress. Three-dimensional principal component analysis (PCA) score plot showing the distribution of individual seminal plasma samples from Zulu rams assigned to four treatment groups: control (blue), selenium supplementation (orange), testicular heat stress (green), and selenium + heat stress (red). Each point represents an individual animal (n = 4 per treatment). The first three principal components explained 55.40% of the total variance in the metabolomic dataset, with PC1, PC2, and PC3 accounting for 30.97%, 15.70%, and 8.73%, respectively.

Principal Component Analysis (PCA) was performed to evaluate the overall variation in seminal plasma metabolites among Zulu rams subjected to selenium supplementation and/or testicular heat stress. The three-dimensional PCA score plot explained 55.40% of the total variation, with PC1 accounting for 30.97%, PC2 for 15.70%, and PC3 for 8.73% of the variance (Figure 3). The PCA revealed a moderate separation among treatment groups, indicating that both selenium supplementation and heat stress altered the seminal plasma metabolomic profile. Samples from the control group generally clustered together and showed positive PC3 scores, suggesting a relatively conserved metabolic profile under normal physiological conditions. In contrast, heat-stressed animals tended to occupy different regions of the PCA space, particularly along PC1 and PC3, indicating that heat stress induced substantial metabolic perturbations.

Selenium-treated animals showed partial separation from the control group, demonstrating that selenium supplementation influenced metabolite composition even in the absence of heat stress. Interestingly, the selenium + heat stress group occupied an intermediate position between the selenium and heat stress groups, suggesting that selenium modified the metabolic response of rams exposed to elevated testicular temperatures. Although some overlap between treatments was observed, reflecting inherent biological variability among animals, distinct clustering patterns indicate treatment-specific metabolic responses. The PCA demonstrates that selenium supplementation and testicular heat stress contribute to measurable changes in the seminal plasma metabolome of Zulu rams and supports the presence of metabolite signatures associated with these treatments.

### 3.4. Variable Importance in Projection (VIP) Score Analysis

**Figure 4:**
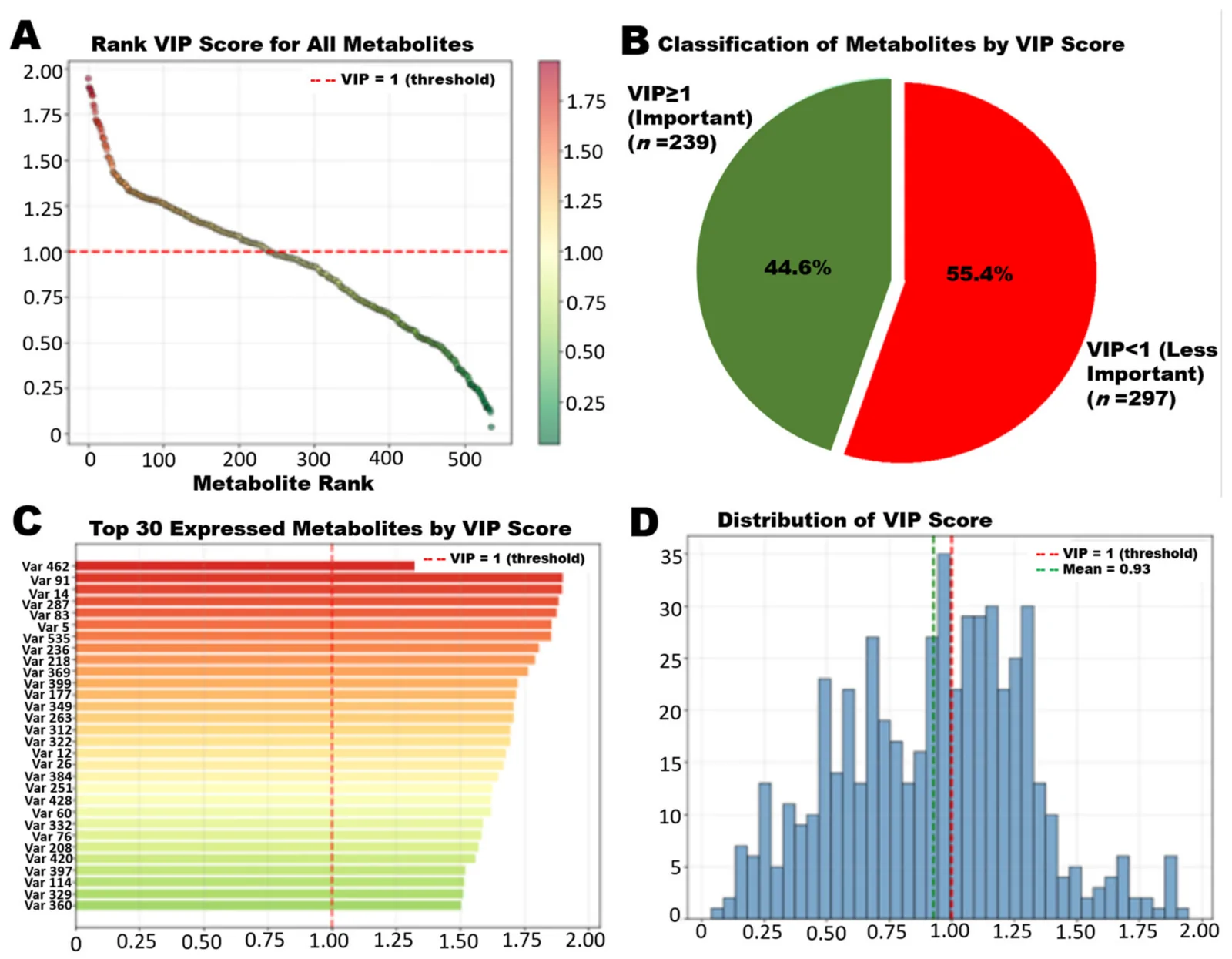
Variable Importance in Projection (VIP) score analysis of metabolites identified by PLS-DA. (**A**) Ranked VIP scores of all 536 metabolites, arranged from highest to lowest, with the red dashed line indicating the conventional importance threshold (VIP = 1.0). (**B**) Classification of metabolites according to the VIP threshold, showing 239 metabolites (44.6%) with VIP ≥ 1.0 (important) and 297 metabolites (55.4%) with VIP < 1.0 (less important). (**C**) Top 30 metabolites ranked by VIP score, highlighting the variables contributing most strongly to group discrimination. (**D**) Frequency distribution of VIP scores across all metabolites. The red dashed line denotes the VIP threshold (1.0), while the green dashed line indicates the mean VIP score (0.93), illustrating the overall distribution of metabolite importance within the PLS-DA model.

The Variable Importance in Projection (VIP) analysis identified metabolites that contributed most significantly to discrimination between the experimental groups. A total of 536 metabolites were evaluated, with VIP scores ranging from 0.0385 to 1.9475 and an average score of 0.9268 ± 0.3760 (mean ± SD). As shown in Figure 4A, VIP scores decreased gradually from the highest- to the lowest-ranked metabolites. Using the conventional threshold of VIP ≥ 1.0, 239 metabolites (44.6%) were classified as important variables, whereas 297 metabolites (55.4%) had VIP scores below the threshold and were considered less influential in explaining group separation (Figure 4B). The highest-ranking metabolites included Var 462 (VIP = 1.948), Var 91 (VIP = 1.899), Var 14 (VIP = 1.897), Var 287 (VIP = 1.882), and Var 83 (VIP = 1.875), indicating that these metabolites contributed most strongly to the predictive PLS-DA model (Figure 4C).

The distribution of VIP scores (Figure 4D) showed a broad range across the metabolite dataset, with the majority of metabolites clustering between 0.7 and 1.3. The mean VIP score (0.93) was slightly below the importance threshold of 1.0, suggesting that while many metabolites contributed moderately to class discrimination, a substantial subset exhibited stronger discriminatory power. Quartile analysis further indicated an even distribution of metabolites across the four VIP score quartiles (134 metabolites per quartile). The upper quartile (VIP > 1.1919) contained the most discriminative metabolites, whereas the lowest quartile (VIP ≤ 0.6499) comprised metabolites with minimal contribution to model performance. Overall, approximately 64% of the metabolites (343 metabolites) accounted for nearly 80% of the cumulative VIP contribution, indicating that the discriminative information was concentrated within a relatively limited subset of the metabolome.

### 3.5. KEGG Pathway Analysis of Selenium-Induced Metabolic Changes in Zulu Rams

**Figure 5:**
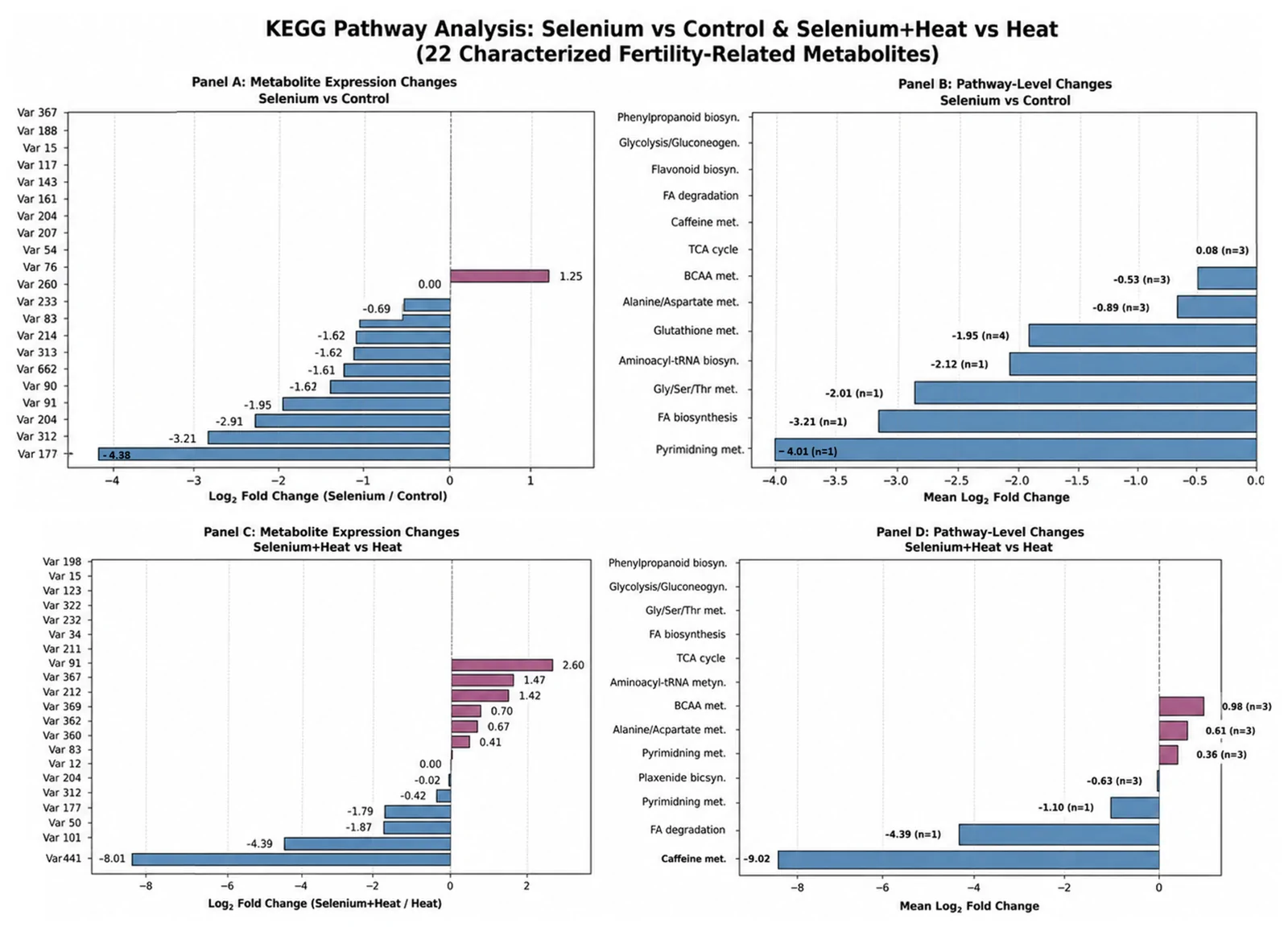
KEGG pathway analysis of characterized fertility-related metabolites in the seminal plasma of Zulu rams following selenium supplementation under normal and heat stress conditions. Panel (**A**) shows metabolite-level expression changes between selenium (Se) and control groups, expressed as log_2_ fold change (Se/control). Panel (**B**) presents the corresponding path-way-level responses, highlighting alterations in pyrimidine metabolism, fatty acid biosynthesis, glycine/serine/threonine metabolism, aminoacyl-tRNA biosynthesis, glutathione metabolism, alanine/aspartate metabolism, BCAA metabolism, and the TCA cycle. Panel (**C**) illustrates metabolite-level changes between selenium + heat stress (SeTHS) and heat stress (THS) groups, expressed as log_2_ fold change (SeTHS/THS). Panel (**D**) shows pathway-level responses, indicating modulation of glutathione metabolism, amino acid metabolism, fatty acid degradation, pyrimidine metabolism, flavonoid biosynthesis, and caffeine metabolism. Positive values indicate increased metabolite abundance, whereas negative values indicate reduced abundance relative to the comparison group. Selenium supplementation altered pathways associated with antioxidant defence, energy metabolism, and cellular homeostasis, particularly under heat stress conditions.

To investigate the biological significance of selenium-induced alterations in the seminal plasma metabolome, characterized fertility-related metabolites were mapped to KEGG pathways and compared between selenium vs. control and selenium + heat stress vs. heat stress treatments (Figure 5). The analysis revealed substantial changes in several metabolic pathways associated with energy metabolism, antioxidant defence, amino acid metabolism, and cellular biosynthesis. Comparison of selenium-supplemented animals with controls identified marked reductions in the abundance of several metabolites, with log_2_ fold-change values ranging from −0.69 to −6.38 (Figure 5A). The greatest decrease was observed for Var177 (log_2_FC = −6.38), followed by Var322 (−3.21), Var236 (−2.91), Var91 (−2.12), Var60 (−1.95), and Var462 (−1.82). In contrast, only one metabolite (Var369) showed an increase in abundance following selenium supplementation (log_2_FC = 1.25).

Pathway-level analysis demonstrated that selenium supplementation primarily affected pyrimidine metabolism, fatty acid biosynthesis, glycine/serine/threonine metabolism, aminoacyl-tRNA biosynthesis, glutathione metabolism, alanine/aspartate metabolism, branched-chain amino acid metabolism, and the tricarboxylic acid (TCA) cycle (Figure 5B). Among these, pyrimidine metabolism exhibited the largest overall reduction (mean log_2_FC = −4.03), followed by fatty acid biosynthesis (−3.21), glycine/serine/threonine metabolism (−2.91), aminoacyl-tRNA biosynthesis (−2.12), and glutathione metabolism (−1.95). Under heat stress conditions, selenium supplementation induced a distinct metabolic response compared with heat stress alone (Figure 5C). Several metabolites increased substantially in abundance, including Var367 (log_2_FC = 2.60), Var218 (1.57), Var287 (1.42), Var462 (0.70), Var369 (0.67), and Var83 (0.41). Conversely, pronounced reductions were observed for Var117 (−8.91), Var161 (−4.39), Var60 (−1.87), and Var177 (−1.79). At the pathway level, selenium supplementation under heat stress enhanced pathways related to branched-chain amino acid (BCAA) metabolism (mean log_2_FC = 0.98), alanine/aspartate metabolism (0.61), and glutathione metabolism (0.36) (Figure 5D). In contrast, pathways associated with caffeine metabolism (−8.91), fatty acid degradation (−4.39), pyrimidine metabolism (−1.10), and flavonoid biosynthesis (−0.03) were reduced relative to heat-stressed animals that did not receive selenium supplementation. These findings demonstrate that selenium supplementation induces significant remodelling of the seminal plasma metabolome and influences multiple metabolic pathways involved in cellular protection, energy utilization, and reproductive function.

## 4. Discussion

The Zulu ram, indigenous to KwaZulu-Natal, faces threats from declining numbers, requiring fertility studies and semen analysis to restore its population [7,12,16]. The current research builds on the understanding of seminal plasma’s complex composition and its pivotal role in fertility, setting the stage for novel investigations into its metabolomic responses to environmental and nutritional factors. Recent advances in multi-omics technologies, as exemplified in research by [17], underscore the importance of metabolomics to unravel the molecular mechanisms underlying meat quality, providing valuable insights into optimizing production traits, which may also be applicable in enhancing fertility biomarkers and metabolic responses in heat-stressed rams. To our knowledge, this study is the first of its kind to explore the impact of selenium on metabolites as fertility biomarkers within the seminal plasma metabolome under induced testicular heat stress in livestock. Expanding upon our recent work, which demonstrated the protective effects of selenium in maintaining sperm quality and testicular parameters in heat-stressed Zulu rams [18], this study aims to further elucidate the metabolic changes associated with selenium and heat stress.

Untargeted GC–TOF/MS metabolomics identified 536 metabolites, of which 359 were conserved across all treatments (Figure 1), indicating the existence of a stable core seminal plasma metabolome in Zulu rams. However, selenium supplementation generated distinct metabolomic signatures, including 53 unique metabolites under normal conditions and 42 unique metabolites under heat stress. The appearance of treatment-specific metabolites suggests that selenium influences biochemical pathways beyond its classical antioxidant role, with metabolite mapping indicating potential alterations in metabolic pathways associated with reproductive physiology and responses to thermal stress rather than direct evidence of pathway activation or functional effects. Multivariate analyses further supported these observations. PCA revealed treatment-dependent clustering patterns, with heat-stressed animals displaying greater separation from the control group than selenium-supplemented animals (Figure 3). This finding suggests that heat stress was a major driver of metabolic variation, consistent with reports showing that thermal stress disrupts energy metabolism, membrane stability, and redox balance in reproductive tissues [19,20]. Interestingly, animals receiving selenium under heat stress occupied an intermediate position between the selenium and heat-stressed groups, indicating that selenium was associated with a partial shift in the metabolomic profile relative to heat stress alone. The VIP analysis further strengthened the evidence for selenium-associated metabolic differences by identifying a subset of metabolites that contributed disproportionately to treatment discrimination (Figure 4). Although many metabolites contributed modestly to group separation, approximately 45% exceeded the VIP threshold of 1.0, indicating that a relatively small proportion of the metabolome accounted for most of the observed biological variation. Such patterns are common in metabolomics studies where a limited number of biologically active metabolites drive physiological responses to nutritional or environmental interventions.

Under normal physiological conditions, selenium supplementation induced metabolites associated with aminoacyl-tRNA biosynthesis, glycine, serine and threonine metabolism, pyrimidine metabolism, and glycolysis/gluconeogenesis (Figure 5; Table 1). Among these, aminoacyl-tRNA biosynthesis was represented by Var 91 and Var 115, both classified as important metabolites (VIP ≥ 1). This pathway plays a critical role in protein synthesis by ensuring accurate amino acid attachment to transfer RNA molecules [21]. Proper translational control is essential for spermatogenesis, sperm maturation, and maintenance of reproductive tissues. The association of these metabolites with aminoacyl-tRNA biosynthesis suggests that selenium supplementation may be accompanied by alterations in metabolic features linked to protein synthesis; however, the present metabolomic data do not demonstrate increased activity of this pathway.

Selenium supplementation also influenced glycine, serine and threonine metabolism through Var 236. This pathway contributes to one-carbon metabolism, which is required for nucleotide synthesis, methylation reactions, and cellular proliferation. Efficient one-carbon metabolism is particularly important in reproductive tissues where rapid cellular differentiation and renewal occur [22]. In seminal plasma, these amino acids are associated with sperm membrane integrity, energy metabolism, and protection against oxidative stress [23]. Therefore, the identification of metabolites mapping to this pathway is consistent with possible alterations in one-carbon metabolism, although functional consequences cannot be inferred from metabolite profiling alone. Another notable finding was the enrichment of pyrimidine metabolism represented by Var 177. Pyrimidines are essential components of DNA and RNA synthesis and play a central role in cell proliferation and tissue maintenance [24]. Pyrimidine metabolites in seminal plasma may support or regulate sperm energy use and nucleotide balance [25]. Accordingly, these metabolite associations may reflect changes in nucleotide-related metabolism, but they should not be interpreted as evidence that selenium directly regulates these biological processes. Furthermore, the identification of Var 9 within glycolysis/gluconeogenesis pathways indicates that selenium supplementation was associated with metabolites linked to carbohydrate metabolism. Given the high ATP demand of spermatozoa, these observations may warrant further functional investigation but do not demonstrate altered energy metabolism [26].

Under heat stress conditions, selenium supplementation induced additional pathways with mapped metabolites associated with antioxidant protection and cellular resilience. Var 15 was linked to glutathione metabolism, a pathway directly associated with antioxidant defence and sperm quality maintenance. Selenium is a structural component of glutathione peroxidases, which protect spermatozoa against oxidative damage caused by reactive oxygen species [27]. The identification of metabolites mapping to glutathione metabolism is consistent with alterations in metabolic pathways related to antioxidant processes; however, the present study did not measure glutathione peroxidase activity or other functional indicators of antioxidant defence, and therefore pathway activation cannot be concluded. Similarly, Var 224 was associated with ascorbate and aspartate metabolism. Ascorbate is an important non-enzymatic antioxidant involved in scavenging reactive oxygen species and protecting reproductive cells from oxidative injury [28]. The presence of metabolites assigned to this pathway is consistent with potential changes in antioxidant-related metabolism, although the biological significance requires confirmation through targeted biochemical or functional analyses. Energy metabolism was further represented by Var 251, which was assigned to the citrate cycle (TCA cycle). The TCA cycle is a central pathway for ATP generation and is essential for supporting sperm motility and overall reproductive function [29]. The mapping of metabolites to this pathway suggests possible alterations in central carbon metabolism but does not establish increased TCA cycle activity or ATP production. Lipid metabolism also emerged as an important selenium-responsive process through Var 322, which was associated with fatty acid biosynthesis. Fatty acids are key structural components of sperm membranes and influence membrane fluidity, integrity, and fertilizing capacity [30]. Heat stress commonly promotes lipid peroxidation, resulting in membrane damage and reduced sperm viability. The association of metabolites with fatty acid biosynthesis may reflect changes in lipid-related metabolic profiles under selenium supplementation; however, direct effects on membrane stability or protection against lipid peroxidation were not assessed in the present study. It is important to also note that a substantial proportion of selenium-responsive metabolites remained uncharacterized despite exhibiting high VIP scores. Several metabolites, including Var 239, Var 324, and numerous other VIP-associated compounds, could not be assigned to known reproductive pathways using current databases. While this limits biological interpretation, it also highlights the complexity of the seminal plasma metabolome and suggests the existence of previously unrecognized metabolic mechanisms involved in reproductive adaptation. These unidentified metabolites may represent candidate biomarkers associated with selenium supplementation, heat stress, or fertility, although further structural characterization and functional validation are required before biological significance can be established.

## 5. Conclusions

The Zulu sheep breed used in the study is at severe risk of extinction, leading to a limited number of rams allocated per treatment group. The untargeted metabolomic technique proved to be a valuable tool for identifying metabolites in the seminal plasma of Zulu rams, revealing several metabolites that were uniquely induced by selenium supplementation in both the absence and presence of heat. However, a major limitation of the present study is the high proportion of uncharacterized metabolites. This challenge is not unique to the current work but reflects a broader limitation within livestock metabolomics. Compared with human and model organism studies, metabolomic databases for livestock species, particularly indigenous sheep breeds, remain underdeveloped. Consequently, many metabolites detected by untargeted metabolomics cannot be confidently annotated due to limited reference spectra, incomplete pathway databases, and the possibility that some compounds represent species-specific or previously undescribed metabolites. Furthermore, seminal plasma is a highly complex biological matrix containing metabolites derived from multiple reproductive tissues, further complicating metabolite identification and functional interpretation. Future studies should focus on the structural elucidation and validation of these unidentified metabolites using complementary analytical approaches such as LC–MS/MS, high-resolution mass spectrometry, NMR spectroscopy, and targeted metabolomics. Integration of metabolomics with transcriptomics, proteomics, sperm functional assessments, and reproductive performance data would further improve pathway annotation and biomarker validation. Such multi-omics approaches may uncover novel metabolic mechanisms underlying fertility and heat-stress adaptation in indigenous livestock. Overall, this study establishes a foundational metabolomic resource for Zulu ram reproductive biology and provides new insights into the metabolic pathways potentially involved in selenium-mediated protection against heat stress. The findings contribute to the growing field of livestock reproductive metabolomics and provide a basis for future biomarker discovery and nutritional strategies to improve fertility and climate resilience in indigenous sheep breeds.

## Figures and Tables

**Table 1:** The metabolites induced under selenium supplementation in the semen of Zulu rams are grouped according to their class and functions related to fertility.

Entry	Class	Classification	KEGG_Pathways	Function in Fertility
**Selenium-induced unique metabolites**				
Var 9	Carbohydrate Metabolism	Important (VIP ≥ 1)	Glycolysis/Gluconeogenesis	Metabolic competition during reproduction
Var 12	Metabolic signal	Important (VIP ≥ 1)	Glycolysis/Gluconeogenesis	ATP generation
Var 239	Uncharacterized	Important (VIP ≥ 1)	Carbon fixation	Not yet characterized in reproductive literature
Var 118; Var 120; Var 135; Var 183; Var 227; Var 266; Var 283; Var 300; Var 304	Uncharacterized	Important (VIP ≥ 1)	Not in KEGG database	Not yet characterized in reproductive literature
Var 40; Var 113; Var 119; Var 121; Var 139; Var 144; Var 154; Var 160; Var 169; Var 197; Var 199; Var 213; Var 215; Var 226; Var 228; Var 234; Var 240; Var 246; Var 279; Var 286; Var 288; Var 298; Var 307; Var 311; Var 318; Var 328	Uncharacterized	Less Important (VIP < 1)	Not in KEGG database	Not yet characterized in reproductive literature
**Selenium induced under heat stress**				
Var 15	Antioxidant	Important (VIP ≥ 1)	Glutathione metabolism	Sperm quality maintenance
Var 27	Amino Acid (GST)	Important (VIP ≥ 1)	Glycine, serine and threonine metabolism	Cell proliferation control
Var 224	Antioxidant	Important (VIP ≥ 1)	Ascorbate and aspartate metabolism	Improves sperm quality
Var 251	Energy Metabolism	Important (VIP ≥ 1)	Citrate cycle (TCA cycle)	Bioenergetics
Var 322	Lipid Metabolism	Important (VIP ≥ 1)	Fatty acid biosynthesis	Membrane lipid composition
Var 324	Uncharacterized	Important (VIP ≥ 1)	Sphingolipid metabolism	Not yet characterized in reproductive literature
Var 72; Var 284; Var 285; Var 299; Var 338; Var 445	Uncharacterized	Important (VIP ≥ 1)	Not in KEGG database	Not yet characterized in reproductive literature
Var 2; Var 121; Var 131; Var 141; Var 149; Var 191; Var 200; Var 202; Var 209; Var 217; Var 226; Var 232; Var 234; Var 245; Var 246; Var 249; Var 298; Var 323; Var 339; Var 425; Var 498; Var 534	Uncharacterized	Less Important (VIP < 1)	Not in KEGG database	Not yet characterized in reproductive literature

## Data Availability

The raw data supporting the conclusions of this article will be made available by the authors on request.

## Abbreviations

The following abbreviations are used in this manuscript:

SeTHS	Selenium plus testicular heat stress
Se	Selenium
THS	Testicular heat stress
CASA	Computer Aided Sperm Analyser
